# Viper-induced Consumptive Coagulopathy from a Decapitated Eastern Diamondback Rattlesnake

**DOI:** 10.7759/cureus.6575

**Published:** 2020-01-06

**Authors:** Nicholas V Titelbaum, Samyr Elbadri, James L Wilson, Bobby Desai, Michael Falgiani

**Affiliations:** 1 Emergency Medicine, Ocala Regional Medical Center, University of Central Florida College of Medicine, Ocala, USA

**Keywords:** viper-induced consumptive coagulopathy, crotalidae polyvalent immune fab, rattlesnake, crotalid

## Abstract

We present the case of a patient who presented with viper-induced consumptive coagulopathy after a bite on the thumb from a decapitated eastern diamondback rattlesnake. The patient was evaluated and treated in the Emergency Department and was admitted to the intensive care unit.

## Introduction

Eastern diamondback rattlesnakes are among the largest and most venomous rattlesnakes, and their bites likely have the highest rate of mortality compared with any rattlesnake in the United States [1-2]. Together with the western diamondback rattlesnake, they are responsible for 95% of snakebite fatalities in the United States [3]. The eastern diamondback rattlesnake is native to pine woods, palmetto stands, fields, and dunes stretching from North Carolina to the entirety of peninsular Florida west to Mississippi [1].

Eastern diamondback rattlesnakes are classified as a type of pit viper (or crotalid), with venom injected 75-80% of the time, whereas the remainder of the bites are dry, meaning that no venom was injected as delivery of venom is voluntary [2]. In the United States, an estimated 1,989 nonfatal rattlesnake bites occur every year, and an average of five people die from snake bites in the country annually [4-5]. Untreated eastern diamondback rattlesnake bites have a fatality of 10-20% [6]. In 2017, 753 rattlesnake envenomations were recorded, with 698 treated in a healthcare facility; of these patients, 13 had no effects of envenomation, 199 had minor adverse effects, 370 experienced moderate adverse effects, 56 had severe effects but survived, and 1 person died [7].

## Case presentation

A ­­­­58-year-old male presented to our Emergency Department (ED) with a snakebite on the left thumb with mild surrounding erythema. The patient came across the snake in a field at night and killed the snake by decapitating it. He sustained the bite while handling the dead snake’s head. A friend took photographs of the snake, which confirmed identification of an eastern diamondback rattlesnake-it is the only species of snake to have a rattle and a diamond-shaped pattern on its back in Florida. Prior to arrival to the ED, he had sucked the wound.

The patient was hemodynamically stable but hypertensive on presentation, with a heart rate of 81 beats per minute, respiratory rate of 18 breaths per minute, and blood pressure of 180/82 mm Hg. On physical examination, the patient had two puncture marks on his left thumb, with mild swelling, ecchymosis, and erythema stretching down to the base of the thumb but maintained sensation and full range of motion. He reported tongue numbness, but on examination, there was no tongue swelling or evidence of airway compromise. No other significant findings were noted on examination.

The patient’s medical workup included a complete blood count, complete metabolic panel, magnesium, coagulation panel, fibrinogen, fibrin degradation products, d-dimer, lactate, creatine kinase, troponin, and electrocardiogram (EKG). A radiograph of the left hand was also obtained. Labs were notable for a platelet count of 8,000 per mm^3^ confirmed on repeat analysis, an elevated d-dimer of 3147 ng/mL, low fibrinogen level of 142 mg/dL, and elevated amount of fibrin degradation products at >40 µg/mL. The patient’s partial thromboplastin time (PTT) was decreased (22.7 seconds), and the aspartate aminotransferase (AST) was slightly elevated (38 IU/L). The remaining labs showed no abnormalities. The electrocardiogram showed normal sinus rhythm. No fractures or foreign bodies were noted on the hand X-ray. Knavel's signs were absent on examination; thus, presentation was not clinically consistent with flexor tenosynovitis.

The patient’s labs were concerning for venom-induced consumptive coagulopathy (VICC). The patient was given 1 vial of crotalidae polyvalent immune fab, and per Poison Control Center, labs were rechecked an hour after administration. Repeat platelet level had increased to 84,000 per mm^3^, fibrinogen decreased to 82.0 mg/dL, PTT and increased to 23.6 seconds, and he now had a slightly prolonged PTT of 13.0 seconds and increased international normalized ratio (INR) of 1.15.

The patient was given another 5 vials of crotalidae polyvalent immune fab in the ED as well as 2 units of fresh frozen plasma (FFP) and 1 unit of platelets. He also received 1 mg of lorazepam for mild anxiety and 4 mg of ondansetron for mild nausea. He was also given the Tdap (tetanus/diphtheria/acellular pertussis) vaccine. The patient was admitted to the intensive care unit (ICU) where he received a further 16 units of crotalidae polyvalent immune fab for a total of 22 vials with dosing per Poison Control Center. He also received vancomycin and piperacillin/tazobactam for empiric coverage of methicillin-resistant Staphylococcus aureus (MRSA) and Pseudomonas aeruginosa from the bite. The patient was downgraded from the ICU to the inpatient medicine service 2 days later and was discharged from the hospital the following day. At the time of discharge, labs 30 hours after last crotalidae polyvalent immune fab administration were notable for platelets improved and stable at 112,000 per mm^3^, PTT was 25.7 seconds, PT of 11.8 seconds, INR was 1.04, and fibrinogen was at a normal level of 375.0 mg/dL.

## Discussion

Eastern diamondback rattlesnake envenomation poses the greatest danger compared to any other type of snake east of the Mississippi River. Emergency medicine clinicians should be familiar with the venomous snakes in the region in which they practice as patients may present with photographs or may bring the snake itself. The authors recommend photographs and dissuade against any attempts to kill or capture a snake as these actions increase the likelihood of another bite. Evidence clearly demonstrating that the snake involved was non-venomous will spare the patient from a more extensive workup.

Recognition of a venomous snake should prompt a physician to pursue a further workup corresponding to the effects of the snake’s venom. Pictures of venomous snakes can be found at the Centers for Disease Control website (https://www.cdc.gov/niosh/topics/snakes/types.html). A physician should rely on laboratory evidence to assess the severity of a snake envenomation as patients can be asymptomatic with severe VICC. It is imperative that physicians working in the ED recognize that a bite from a deceased snake can still result in envenomation.

Bites from pit vipers such as eastern diamondback rattlesnakes most commonly present as two-fang punctures with pain and local swelling that develop within five minutes. Bites can also develop surrounding necrosis. Bites in extremities can get infected or develop compartment syndrome. Clinical effects range from asymptomatic and mild local reactions to life-threatening systemic infections such as headache, nausea, vomiting, abdominal pain, diarrhea, and dizziness; less common effects include neurotoxic paralysis, yellow vision, metallic taste, and fasciculations [6].

Eastern diamondback rattlesnake venom is hemotoxic and can cause VICC associated with thrombocytopenia and hypofibrinogenemia. Envenomation can also lead to acute renal failure, hypovolemic shock, and death. Hypotension and shock occur in only 7% of all rattlesnake envenomations [8]. Eastern diamondback venom is hemorrhagic and cytotoxic, with the latter feature sometimes resulting in limb amputation or permanent disability [4]. Coagulopathy is the major clinical effect, and nephrotoxicity and myotoxicity can also occur secondary to the coagulopathy [6]. The venom glands are located in the rattlesnake’s head, and, thus, recently decapitated rattlesnake heads still pose a danger to humans [9].

Thrombocytopenia is a well-described sequela of rattlesnake envenomation, but the exact mechanism remains unknown [10]. The hemotoxic compounds of rattlesnake venom include a combination of procoagulants, a platelet aggregation inhibitor leading to anticoagulation, and zinc metalloproteinases causing hemorrhage [6]. The proteinases contain thrombin-like activity, which depletes circulating fibrinogen by proteolysing its α and β subunits, increasing fibrin degradation products causing prolonged hypofibrinogenemia that can result in hemorrhage [11]. This effect is the etiology of VICC, a condition frequently mistaken for disseminated intravascular coagulation (DIC) in which fibrinolysis is activated by increased levels of endogenous thrombin. While factor replacement is effective in treating DIC, antivenom must be used to inactivate the thrombin-like glycoproteins inducing the coagulopathy caused by rattlesnake venom.

The Poison Control Center should be contacted immediately for diamondback rattlesnake bites. The wound should be cleaned, and tetanus vaccine should be administered if the patient’s vaccination status is unknown or out of date. If envenomation occurs, the edge of swelling should be marked and monitored along with extremity circumference every 30 minutes; if there is no proximal progression and no coagulopathy after 12 hours of observation and serial laboratory evaluations, reliable patients can be discharged home [12-13].

Antivenom is recommended when local injury progresses, a coagulopathy is detected, or systemic effects including hypotension and altered mental status develop. The antivenom of choice for pit vipers such as eastern diamondback rattlesnakes is the ovine crotalidae polyvalent immune fab. Though crotalidae polyvalent immune fab is less allergenic than the formerly used equine Antivenin (Crotalidae) Polyvalent, epinephrine and H1 and H2 receptor blocking antihistamines should be present at bedside when administering crotalidae polyvalent immune fab [3]. Crotalidae polyvalent immune fab is administered as a large initial dose of 4-6 vials over one hour to control envenomation. If there is evidence of progression of symptoms such as increased swelling or systemic bleeding, another 4-6 vials should be administered over one hour, and this dose should be repeated hourly as needed until initial control is achieved. After symptoms are controlled, maintenance dosing of 2 vials every 6 hours for 18 hours is recommended for patients with evidence of coagulopathy and with severe clinical features [3,14]. Though the administration of platelets and FFP might appear indicated based on laboratory abnormalities occurring in VICC, these should be avoided unless there is life-threatening bleeding because the definitive treatment is antivenin [14].

While swelling and erythema at bite sites may appear similar to a skin infection, these are effects of the venom itself and prophylactic antibiotics are not indicated as rattlesnake bites have a very low likelihood of infection due to the proteolytic properties of snake venom [14]. Compartment syndrome is a rare complication of rattlesnake envenomation. Prophylactic fasciotomies can be more harmful than beneficial; thus, it is preferred to monitor for symptoms of compartment syndrome instead [3].

## Conclusions

Prompt recognition of venomous snake bites is imperative in order to provide timely care. The antivenom of choice for pit vipers such as eastern diamondback rattlesnakes is crotalidae polyvalent immune fab. As with all toxic exposures, the Poison Control Center should be contacted immediately for diamondback rattlesnake bites. As with all wounds, thorough cleansing and tetanus prophylaxis should be administered.
